# Nitric oxide synthase inhibition decreases tolerance to hyperoxia in newborn rats

**DOI:** 10.1155/S096293519500069X

**Published:** 1995-11

**Authors:** M. R. Pierce, C. A. Voelker, I. R. S. Sosenko, S. Bustamante, S. M. Olister, X.-J. Zhang, D. A. Clark, M. J. S. Miller

**Affiliations:** 1 Department of Pediatrics Tulane Medical Center New Orleans LA USA; 2 Department of Pediatrics Louisiana State University Medical Center New Orleans LA USA; 3 Department of Pediatrics University of Miami Medical Center Miami FL USA

## Abstract

We evaluated the effects of sustained perinatal inhibition of NO synthase (NOS) on hyperoxia induced lung injury in newborn rats. N^G^-nitro-Larginine-methyl-ester (L-NAME) or untreated water was administered to pregnant rats for the final 7 days of gestation and during lactation; followed by postnatal exposure to hyperoxia (>95% O_2_) or room air. The survival rate of L-NAME treated pups when placed in > 95% O_2_ at birth was significantly lower than controls from day 4 (L-NAME, 87%; control pups, 100%, p < 0.05) to 14 (L-NAME, 0%; control pups, 53%, p < 0.05). Foetal pulmonary artery vasoconstriction was induced by L-NAME with a decrease in internal diameter from 0.88 ± 0.03 mm to 0.64 ± 0.01 mm in control *vs*. L-NAME groups (p < 0.05), respectively. We conclude that perinatal NOS inhibition results in pulmonary artery vasoconstriction and a decreased tolerance to hyperoxia induced lung injury in newborn rats.


Mediators of Inflammation 4, 431–436 (1995)

### Nitric oxide synthase inhibition decreases tolerance to hyperoxia in newborn rats

M. R. Pierce,[1,CA] C. A. Voelker,[2] I. R. S. Sosenko,[3] S. Bustamante,[2] S. M. Olister,[2] X.-J. Zhang,[2] D. A. Clark[2] and M. J. S. Miller[2]

[1]Department of Pediatrics, Tulane Medical Center, New Orleans, LA; [2]Department of Pediatrics, Louisiana State University Medical Center, New Orleans, LA; and [3]Department of Pediatrics, University of Miami Medical Center, Miami, FL, USA

[CA]Corresponding Author

**WE evaluated the effects of sustained perinatal inhibition of NO synthase (NOS) on hyperoxia induced lung injury in newborn rats. $N^G$-nitro-L-arginine-methyl-ester (L-NAME) or untreated water was administered to pregnant rats for the final 7 days of gestation and during lactation; followed by postnatal exposure to hyperoxia (>95% $O_2$) or room air. The survival rate of L-NAME treated pups when placed in >95% $O_2$ at birth was significantly lower than controls from day 4 (L-NAME, 87%; control pups, 100%, $p < 0.05$) to 14 (L-NAME, 0%; control pups, 53%, $p < 0.05$). Foetal pulmonary artery vasoconstriction was induced by L-NAME with a decrease in internal diameter from $0.88 \pm 0.03$ mm to $0.64 \pm 0.01$ mm in control *vs.* L-NAME groups ($p < 0.05$), respectively. We conclude that perinatal NOS inhibition results in pulmonary artery vasoconstriction and a decreased tolerance to hyperoxia induced lung injury in newborn rats.**

**Key words:** Antioxidant enzyme, hyperoxia, $N^G$-nitro-L-arginine-methyl-ester, nitric oxide, nitric oxide synthase, pulmonary artery

### Introduction

Nitric oxide produced in the endothelium plays an important role in regulating vascular tone by activating soluble guanylate cyclase, which leads to smooth muscle relaxation through the synthesis of guanosine 3′,5′-cyclic monophosphate (cGMP).[1] The pulmonary circulation's release and response to NO,[2,3] has been shown to result in potent pulmonary vasodilator in foetal lambs.[3] It also modulates pulmonary vascular tone during the early neonatal period.[2] NO has been recognized as a contributor to the decrease in pulmonary artery pressure that is crucial for a neonate's successful transition from an intrauterine to an extrauterine environment.[4] In addition to its role in the foetal and transitional neonatal circulation, it has also been shown to improve systemic arterial oxygenation in adults with lung injury.[5] Inhibition of endogenous NO formation or release decreases the production of NO by the intact lung and enhances hypoxic pulmonary vasoconstriction.[6] NO appears to play a major role in vascular tone during foetal, transitional and neonatal development.

Hyperoxia upsets the normal cellular oxidant–antioxidant defence equilibrium by producing marked increases in $O_2$ free radical production.[7] This process ultimately results in endothelial cell injury and a hypoxic state.[7] Most studies with inhibitors of NOS are acute studies as opposed to sustained chronic administration of NOS inhibitors. In the present study we explored the ability of the newborn rat to withstand a hyperoxic challenge and its associated endothelial cell injury following chronic perinatal inhibition of NOS with L-NAME, as well as foetal pulmonary artery structure and development. We hypothesized that the stress of hyperoxia to the pulmonary endothelium combined with chronic inhibition of NOS with L-NAME would have deleterious effects on the developing neonatal rat.

### Materials and Methods

*Animals and treatment:* Timed-pregnant rats (Holtzman, Harlan Sprague–Dawley, Indianapolis) were obtained on the 13th day of a 22-day gestation (Fig. 1). Following a day of acclimatization the dams were randomly assigned to one of two treatment groups, either a control group or a L-NAME treatment group. In a rat the 14th day of gestation represents the beginning of the last third of pregnancy. We chose to begin NO blockade at this time since in previous work[8–10] we have demonstrated consistent foetal effects with NO blockade during the last third of pregnancy. L-NAME (Sigma Chemical Co., St. Louis, MO), was administered in the drinking

© 1995 Rapid Science Publishers

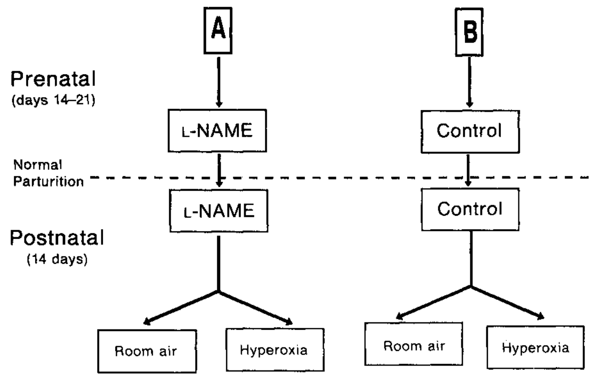

**FIG. 1. Prenatal and postnatal treatment scheme. Dams in Group A received L-NAME (1.0 mg/ml) prenatally throughout the last third of pregnancy (beginning on the 13th day of a 22 day gestation) and throughout the post-natal treatment period (14 days), in drinking water. Following delivery by normal parturition the pups from equivalently treated (L-NAME and control), newly delivered litters were randomly assigned to either a hyperoxia exposure (>95% $O_2$) or room air exposure group.**

water, 1 mg/ml, which was made up fresh daily. The control group received drinking water alone. With little variability, both L-NAME and control dams consumed approximately 70 ml water per day. This amount calculates to a dose of ~180 mg/kg/day. The dose range used in this study, encompassed doses known to reduce the elevated nitric oxide production associated with experimental inflammatory bowel disease,[11,12] and many reports evaluating hypertension induced by chronic reductions of NOS.[12–14] The oral route of administration has been routinely used by us[11] and others[13,14] and is a simple and reliable approach to the chronic administrations of NOS inhibitors.

Newborn rats were obtained by normal parturition within 6–12 h of delivery of the first pup. The newborn pups from several equivalently treated litters (L-NAME or control) were first pooled and then randomly redistributed to the equivalently treated, newly delivered dams. Dams plus ten to twelve pups/litter were randomly assigned to either a hyperoxic exposure (>95% $O_2$) or room air exposure group.

Exposures to hyperoxia (96–98%) were conducted in 3.5-cubic-foot clear plastic exposure chambers. The chambers were opened daily (10–15 min) to provide fresh food and water, to weigh the rat litters and to interchange mothers between litters exposed to $O_2$ and room air, to avoid $O_2$ toxicity in the nursing dams. The offspring were either maintained in hyperoxia or room air for 14 days for survival studies, or killed with an overdose of ketamine–xylazine anaesthesia after 5 days of either hyperoxia (>95% $O_2$) or room air exposure, for the lung analyses described below. All treatment and surgical protocols were approved by the Institutional Animal Care and Use Committee of the Louisiana State University Center in New Orleans, in accordance with guidelines of the Declaration of Helsinki and the National Institutes of Health. Rats were housed in a facility accredited by the American Association for Accreditation of Laboratory Animal Care.

*Lung analysis:* For the analysis of lung antioxidant enzyme (AOE) activity after 5 days of >95% $O_2$ or room air exposure, newborn pups from each group were sacrificed following an overdose of ketamine–xylazine anaesthesia, and their lungs were immediately perfused *in situ* with ice-cold saline and then homogenized in cold saline (20–30:1; v/w). Two to three lungs were pooled per sample to provide adequate lung tissue for the assays. The homogenates were frozen at −70°C for subsequent AOE analyses. The activities of total superoxide dismutase,[15] catalase,[16] and glutathione peroxidase[17] were assayed by standard spectrophotometric techniques. Purified enzyme standards (superoxide dismutase and catalase) were obtained from Sigma Chemical, and glutathione peroxidase standard was obtained from Boehringer–Mannheim Co. (Indianapolis, IN). Lung protein was determined with purified bovine albumin (Sigma) as a standard according to the method of Schacterle,[18] and lung DNA was determined with purified calf thymus DNA (Sigma) as a standard according to the method of Richards.[19]

*Histological evaluation:* Microscopic studies were carried out to evaluate lung structure. For microscopic studies lungs were inflated *in situ* through a tracheal catheter at a constant 20 cm $H_2O$ pressure (fixative, 10% buffered formalin). Fixation was continued at room temperature for 48 h before sectioning. From all lungs, similarly oriented sections from similar portions of the left lung were stained with haematoxylin and eosin.

Pulmonary oedema was microscopically assessed in coded lung sections from evidence of interstitial or peribronchial–perivascular swelling and eosinophilic-positive staining (proteinaceous) material within the air spaces (intra-alveolar oedema). Pulmonary oedema was also assessed by comparative wet/dry lung weight ratios using non-perfused lung lobes weighed before and after drying in an 80°C oven for 48 h to reach constant weight.

*Foetal pulmonary artery diameter:* In a subgroup of L-NAME and control animals, at gestational day 21, dams were anaesthetized, foetuses removed by hysterotomy and rapidly frozen in

**Table 1.** Comparative parameters in L-NAME and control rats offspring following 5 days in >95% $O_2$ or room air*

| Treatment group | Body wt (g) | Lung wt (g) | Lung wt/body wt (%) | Protein (mg/g lung) | DNA (mg/g lung) | Protein/DNA |
|---|---|---|---|---|---|---|
| Air control | 13.82 ± 0.45† | 0.225 ± 0.006 | 1.67 ± 0.04 | 153.41 ± 1.94 | 8.07 ± 0.29 | 19.25 ± 0.85 |
| Air L-NAME | 10.38 ± 0.49 | 0.208 ± 0.017¶ | 1.93 ± 0.09 | 151.89 ± 2.75 | 7.78 ± 0.47 | 20.05 ± 0.98 |
| $O_2$ control | 12.67 ± 0.56 | 0.198 ± 0.012 | 1.53 ± 0.10 | 152.92 ± 3.19 | 6.22 ± 0.43‡ | 25.20 ± 1.58‡ |
| $O_2$ L-NAME | 9.19 ± 0.30§ | 0.154 ± 0.007§ | 1.66 ± 0.05 | 149.08 ± 2.01 | 6.71 ± 0.50 | 23.37 ± 1.78 |

*Values are mean ± 1 S.D. for six to eight litters per group.
†Statistically significant with $p < 0.05$ for air L-NAME *vs.* air control.
§Statistically significant with $p < 0.05$ for $O_2$ L-NAME *vs.* $O_2$ control.
‡Statistically significant with $p < 0.05$ for $O_2$ control *vs.* air control.
¶Statistically significant with $p < 0.05$ for $O_2$ L-NAME *vs.* air L-NAME.

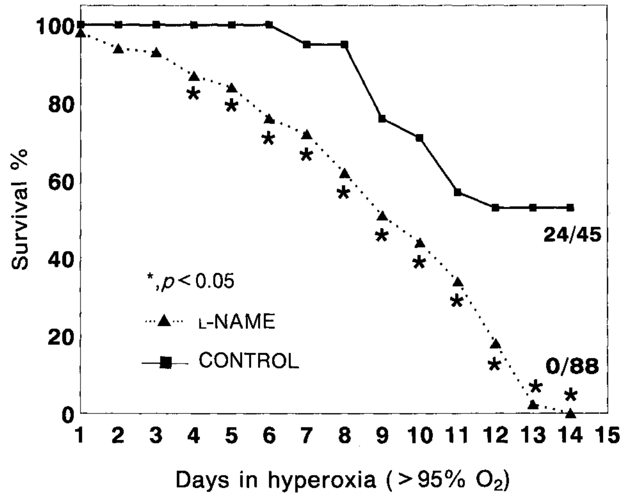

FIG. 2. Survival of L-NAME-treated and control newborns in >95% $O_2$ for 14 days (*n*/*n* = number alive/number placed in $O_2$). The L-NAME treated pup survival rate is significantly decreased compared with $O_2$ controls at all time periods from 4 to 14 days in hyperoxia. *$p < 0.05$, Chi-square test.

isopentane chilled with liquid nitrogen (< −150°C). Cryostat sections 10 μm thick were mounted and stained on microscopic slides. As described previously[20] an imagery computer program was then used to reconstruct the pulmonary artery using 3D imaging techniques. This computer technology allows measurements of the internal diameters of the pulmonary arteries with electronic calipers.

*Statistical analysis:* Survival rates of the treated *vs.* untreated rat pups and assessment of intra-alveolar edema were compared by Chi-square testing.[21] For comparing biochemical values for the two hyperoxic groups with those of the two air control groups, one-way analysis of variance was done, followed by Duncan's multiple range test.[21] For foetal pulmonary artery diameters, one-way analysis of variance was done followed by the Student–Newman–Keuls test. For all statistical tests, a $p < 0.05$ value was considered to represent a significant difference between the compared values.

### Results

*Physical characteristics:* The influence of prenatal L-NAME on pup body weight was determined at birth, and postnatal L-NAME influence was determined after 5 days in air and hyperoxia. Body weights were significantly decreased at birth in the L-NAME treated offspring (6.08 ± 0.40) compared with control offspring (7.45 ± 0.10, $p < 0.05$). (In previous reports we have addressed the intrauterine foetal growth retardation associated with prenatal L-NAME administration[8]). Consistent with the reduction in pup size is a time dependent reduction in placental weight. We have speculated that inhibition of endothelial-derived NO may compromise placental function limiting the maternal–foetal exchange of oxygen, nutrients and metabolic wastes.

The pups exposed to L-NAME had weights which remained significantly decreased after 5 days in air (−25%) or in hyperoxia (−27%) (Table 1) ($p < 0.05$). Lung wet weights were also significantly decreased after 5 days in hyperoxia in the L-NAME (−23%) treated offspring, but this was in proportion to the reduction in body weight, as the ratio of lung weight to body weight was unaltered (Table 1). L-NAME treatment followed by 5 days in air or hyperoxia did not result in significant changes in the normal rate of increase of lung protein or DNA content associated with the neonatal maturation process. No differences were noted in the protein/DNA ratio with L-NAME administration although there was a tendency for this ratio to increase with hyperoxia (Table 1).

*Survival data:* The offspring perinatally treated with L-NAME demonstrated a significantly decreased survival rate compared to the control offspring from the 4th day onward in hyperoxia, with the comparative 13 day survival rate being >20 times less (2.3%) for the $O_2$ L-NAME group *vs.* the $O_2$ control group (53%) (Fig. 2). The survival rates for the room air L-NAME and control groups were 96% and 100%, respectively.

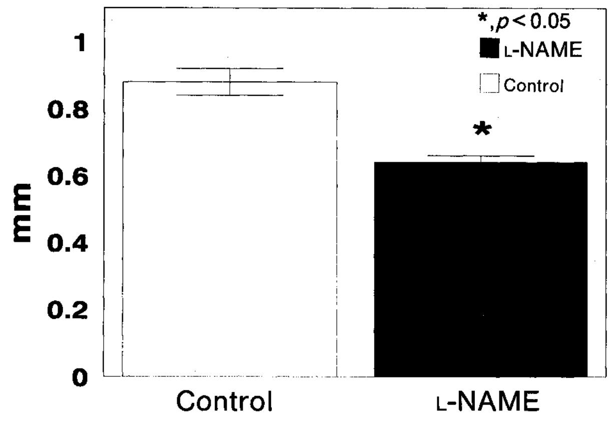

FIG. 3. Pulmonary artery internal diameters for L-NAME and control pups, $n=7$. The L-NAME treated pups had a significant decrease in pulmonary diameter compared with $O_2$ control. $^*p < 0.05$, Student-Newman-Keuls test.

**Table 2.** Comparative pulmonary antioxidant enzyme activities in L-NAME and control offspring after 5 days in >95% $O_2$ room air*

| Treatment group | SOD | CAT | GP |
|---|---|---|---|
| Air control | 106.4 ± 4.5 | 127.9 ± 7.5 | 0.591 ± 0.103 |
| Air L-NAME | 101.9 ± 2.0 | 149.3 ± 6.4¶ | 0.713 ± 0.026¶ |
| $O_2$ control | 123.1 ± 7.5‡ | 261.3 ± 24.1‡ | 1.066 ± 0.046‡ |
| $O_2$ L-NAME | 112.4 ± 2.4 | 296.6 ± 23.8 | 1.218 ± 0.047§ |

*Values are means ± 1 S.D. for six to eight litters, 14 to 15 samples per group. Antioxidant enzymes: superoxide dismutase (SOD), catalase (CAT), and glutathione peroxidase (GP) are expressed as activity units/mg DNA.
§Statistically significant with $p < 0.05$ for $O_2$ L-NAME *vs.* $O_2$ control.
‡Statistically significant with $p < 0.05$ for $O_2$ control *vs.* air control.
¶Statistically significant with $p < 0.05$ for $O_2$ L-NAME *vs.* air L-NAME.

*Pulmonary artery diameter:* Pulmonary artery internal diameters were significantly reduced in L-NAME treated *vs.* control foetuses at 21 days of gestation (Fig. 3).

*Lung biochemistry:* Comparative pulmonary AOE activity responses after 5 days of hyperoxia were similar between the $O_2$–L-NAME *vs.* $O_2$–control groups (Table 2). Following a hyperoxic challenge both L-NAME and control groups demonstrated a significant increase in CAT (99% and 104%, respectively) and GP (71% and 80%, respectively) following hyperoxic challenge (Table 2).

*Histological studies:* Qualitative examination revealed that all the $O_2$-exposed pups (from both L-NAME and control groups) had evidence of perivascular or peribronchiolar oedema present after 5 days of hyperoxic exposure. This microscopic finding was further substantiated by the increased wet/dry lung weights of the $O_2$-exposed offspring *vs.* the offspring maintained in room air from both groups. After 5 days in hyperoxia, no differences were observed in wet/dry weights between the L-NAME (5.95 ± 0.54) and the control (6.08 ± 0.48) offspring (average air control = 5.51 ± 0.06). L-NAME did not appear to alter the dysmorphology associated with sustained hyperoxia (atelectasis, edema).

### Discussion

Mechanisms that regulate vascular tone, growth and function in the developing pulmonary circulation are incompletely understood.[22] Acetylcholine, which putatively causes vascular relaxation through the release of endogenous NO,[23] is a potent pulmonary vasodilator in the foetal lamb.[3] Following birth, it appears that endogenous NO continues to be a key modulator of pulmonary vascular tone in the lamb.[24] Recent studies have demonstrated that the reduction of pulmonary vascular resistance after the onset of air breathing is partly caused by the release of NO from the endothelium.[25] Located between the circulation and underlying smooth muscle, the endothelial cell plays a central role in producing changes in its physical, chemical and neurohumoral environment through the production and release of vasodilators and vasoconstrictors. At birth, the release of NO appears to be triggered by an increase in pulmonary oxygen tension, blood flow and mechanical stretch of the lung.[25] Inhibition of endogenous NO formation or release attenuates the reduction of pulmonary vascular resistance and the elevation in pulmonary artery blood flow associated with the initiation of air breathing by the lamb.[6] In rats we have previously reported a significantly higher basal NO release in newborn femoral vessels *vs.* adult femoral vessels,[9] suggesting that up-regulation of basal NO release is increased throughout the systemic circulation of the newborn compared to the adult.

Beyond its role in the prenatal and transitional circulation, NO also modulates pulmonary vascular tone during the early neonatal period.[2,24] In a previous report we have also shown an increase in systemic vascular resistance, measured by carotid artery cannulation in 21-day old rat pups following chronic perinatal L-NAME exposure.[10] Blockade of NO has been shown to augment smooth muscle proliferation and the release of vasoactive agents in isolated vascular arteries and cell culture systems.[10]

Hyperoxia upsets the normal cellular oxidant–antioxidant defence equilibrium by producing marked increases in $O_2$ free radical production.[7] This process results in endothelial cell injury and a hypoxic state. We explored the ability of newborn rats to withstand a hyperoxic challenge following chronic perinatal administration of the NOS inhibitor, L-NAME. We hypothesized that the stress of hyperoxia to the developing newborn pulmonary endothelium combined with chronic perinatal inhibition of NOS would not be well tolerated. As we anticipated the offspring treated perinatally with L-NAME demonstrated a significantly decreased survival rate compared to the control offspring from the 4th day onward in hyperoxia, with the comparative 13 day survival rate being >20 times less for the $O_2$–L-NAME group versus the $O_2$–control group (Fig. 1). Thus, we have demonstrated possible pulmonary deleterious effects of perinatal NOS inhibition in the rapidly growing neonatal lung exposed to hyperoxia.

The superior ability of newborn infants to resist $O_2$-induced lung damage (and lethality) compared with adult animals is at least partly related to the newborn's ability to increase its basal AOE activity levels in response to hyperoxia, a biochemical response adult animals do not demonstrate.[26–29] We evaluated whether perinatal NOS inhibition would negate a neonate's ability to mount a protective AOE response to a hyperoxic challenge. Our comparative AOE levels at 5 days of life were not different between the L-NAME and control pups exposed to hyperoxia (Table 2). Hence, chronic perinatal inhibition of NOS does not alter the neonatal rat's ability to mount an AOE response to a hyperoxic challenge and does not account for the hyperoxic lethality in this study. Also, we were not able to find significant differences in other measures of $O_2$ radical-induced lung injury, including lung weight/body weight ratio, DNA or protein content or protein/DNA ratio in lung tissue (Table 1), pulmonary oedema (microscopically or by wet/dry weights) or microscopic evidence of tissue destruction.

However, near term (21st day of a 22 day gestation), the internal diameters of pulmonary arteries from L-NAME treated foetuses were significantly smaller than those in control foetuses (Fig. 2). The distinct mortality data suggests that L-NAME treatment may have sensitized newborn rats to the lethal effects of hyperoxia via its effects on the pulmonary vasculature. Pulmonary vasoconstriction *per se* (L-NAME treated neonatal rats exposed to air) did not cause lethality. Our findings are in agreement with the work of Kourembanas *et al.*, who report that inhibition of NO augments smooth muscle proliferation and the release of vasoactive agents in isolated vascular arteries and cell culture systems.[30] The neonatal pulmonary circulation can rapidly undergo structural remodelling, leading to altered vessel diameter and compliance resulting from smooth muscle cell proliferation. Our current and previous works merge with the Kourembanas studies and suggest that inhibition of NO may alter pulmonary vascular tone and structure. These changes may result from a decreased release of vasodilator substances, an increased release of vasoconstrictors, and an altered smooth muscle cell responsiveness to vasoactive mediators, or by structural remodelling of the pulmonary vessels.

In summary, basal NO release from endothelial cells is increased throughout the systemic circulation of the newborn compared to the adult[9] and is a key modulator of pulmonary vascular tone during the transition from foetus to newborn[24] and during the early newborn period.[2,24] NOS inhibition decreases the internal diameter of the pulmonary arteries, increases pulmonary vascular resistance[6] and decreases pulmonary artery blood flow.[6] The summation of these pulmonary changes, compounded by hyperoxia induced endothelial cell injury, significantly alters NO production and release to the extent that mortality is significantly influenced. Further study into the ontogeny of the L-arginine:NO pathway will improve our understanding of how the transition from foetus to neonate is executed, neonatal development, and potentially target strategies for remedying damage induced to a developing neonate from a hyperoxic exposure (i.e. bronchopulmonary disease and retinopathy of prematurity).

### References

1. Holzmann S. Endothelium-induced relaxation by acetylcholine associated with larger rises in cGMP in coronary arterial strips. *J Cyclic Nucleotide Res* 1982; **8:** 409–419.
2. Davidson D, Eldemerdash A. Endothelium-derived relaxing factor: presence in pulmonary and systemic arteries of the newborn guinea pig. *Pediatr Res* 1990; **27:** 128–132.
3. Cassin S, Dawes GS, Mott JC, *et al.* The vascular resistance of the foetal and newly ventilated lung of the lamb. *J Physiol* 1966; **171:** 61–79.
4. Abman SH, Chatfield BA, Hall SL, McMurtry IF. Role of endothelium-derived relaxing factor during transition of pulmonary circulation at birth. *Am J Physiol* 1990; **259:** H1921–H1927.
5. Rossaint F, Falke KJ, Lopez F. Inhaled nitric oxide in adult respiratory distress syndrome. *N Engl J Med* 1993; **328:** 399–405.
6. Rees DD, Palmer RMJ, Hodson HF, Moncada S. A specific inhibitor of nitric oxide formation from L-arginine attenuates endothelium-dependent relaxation. *Br J Pharmacol* 1989; **96:** 418–424.
7. Zweir JL, Duke SS, Kuppusamy P, Sylvester JT, Gabrielson EW. Electron paramagnetic resonance evidence that cellular oxygen toxicity is caused by generation of superoxide and hydroxyl free radical. *FEBS Lett* 1989; **252:** 12–16.
8. Diket AL, Pierce MR, Munshi UK, Voelker CA, Eloby-Childress S, Greenberg S, Zhang X-J, Clark DA, Miller MJS. Nitric oxide inhibition causes intrauterine growth retardation and hind-limb disruptions in rats. *Am J Obstet Gynecol* 1994; **171:** 1243–1250.
9. Pierce RL, Pierce MR, Voelker CA, Miller MJS. Endothelium dependent limb reduction defects following prenatal inhibition of nitric oxide synthase in rats. *Pediatr Res* 1995; **37:** 389A.
10. Voelker CA, Miller MJS, Zhang Xioa-Jing, Eloby-Childress S, Clark DA, Pierce MR. Perinatal nitric oxide synthase inhibition retards neonatal growth by inducing hypertrophic pyloric stenosis in rats. *Pediatr Res* 1995; **38:** 768–774.

11. Miller MJS, Sadowska-Krowicka H, Chotinaruemol S, Kakkis JL, Clark DA. Amelioration of chronic ileitis by NOS inhibition. *J Pharmacol Exp Ther* 1993; **264:** 11–16.
12. Grisham MB, Specian RD, Zimmerman TE. Effects of nitric oxide synthase inhibition on the pathophysiology observed in a model of chronic granulomatous colitis. *J Pharmacol Exp Ther* 1994; **271:** 1114–1121.
13. Gardiner SM, Compton AM, Bennett T, Palmer RMJ, Moncada S. Regional hemodynamic changes during oral ingestion of $N^G$-monomethyl-L-arginine or $N^G$-nitro-L-arginine methyl ester in conscious Brattleboro rats. *Br J Pharmacol* 1990; **101:** 10–12.
14. Baylis C, Mitruka B, Deng A. Chronic blockage of nitric oxide synthesis in the rat produces hypertension and glomerular damage. *J Clin Invest* 1992; **90:** 278–281.
15. McCord JM, Fridovich I. Superoxide dismutase: an enzymatic function for erythocuprein (hemocuprein). *J Biol Chem* 1969; **244:** 6049–6055.
16. Holmes RS, Masters CJ. Epigenetic interconversions of the multiple forms of mouse liver catalase. *FEBS Lett* 1960; **11:** 45–48.
17. Paglia DE, Valentine WN. Studies on the quantitative and qualitative characterization of erythrocyte glutathione peroxidase. *J Lab Clin Med* 1967; **70:** 158–159.
18. Schacterle GR, Pollack RL. A simplified method for the quantitative assay of small amounts of protein in biological materials. *Anal Biochem* 1973; **51:** 654–655.
19. Richards GM. Modification of the diphenylamine reaction giving increased sensitivity and simplicity in the estimation of DNA. *Anal Biochem* 1974; **57:** 369–376.
20. Gasser RF, Shigihara S, Shimada K. Three-dimensional development of the facial nerve path through the ear region in human embryos. *Ann Otol Rhinol Laryngol* 1994; **103:** 395–403.
21. Steel RGD, Torrie JH. *Principles and Procedures of Statistics.* New York: McGraw Hill, 1960; 366–387.
22. Tod ML, Cassin S. Fetal and neonatal pulmonary circulation. In: Crystal R, West JB, eds. *The Lung: Scientific Foundations.* New York: Raven Press, 1991; 1687–1698.
23. Ignarro LJ, Burke TM, Wood KS. Association between cyclic GMP accumulation and acetylcholine-elicited relaxation of bovine intrapulmonary artery. *J Pharmacol Exp Ther* 1983; **228:** 682–690.
24. Fineman JR, Heymann MA, Soifer SJ. *N*-nitro-L-arginine attenuates endothelium-dependent pulmonary vasodilation in lambs. *Am J Physiol* 1991; **260:** H1299–H1306.
25. Cornfield DN, Chatfield BA, McQuestion JA. Effects of birth-related stimuli on L-arginine-dependent pulmonary vasodilation in ovine fetus. *Am J Physiol* 1992; **262:** H1474–H1481.
26. Frank L. Developmental aspects of experimental pulmonary oxygen toxicity. *Free Radic Biol Med* 1991; **11:** 463–494.
27. Franburg BL, Denke SM. Hyperoxia, toxicity and adaptation. In: Massaro D, (ed). *Lung Cell Biology, Vol 41.* (In the series: *Lung Biology in Health and Disease*). New York: Marcel-Dekker, 1989; 1199–1226.
28. Frank L, Sosenko IRS. Failure of premature rabbits to increase antioxidant enzymes during hyperoxic exposure: increase susceptibility to pulmonary oxygen toxicity compared with term rabbits. *Pediatric Res* 1991; **29:** 292–296.
29. Roberts RJ, Frank L. Developmental consequences of oxygen toxicity. In: Kacew S, Reasor M, (eds). *Toxicology and the Newborn.* New York: Elsevier Scientific, 1984; 141–171.
30. Kourembanas S, McQuillan LP, Leung GK. NO regulates the expression of vasoconstrictors and growth factors by vascular endothelium under both normoxia and hypoxia. *J Clin Invest* 1993; **92:** 99–104.

ACKNOWLEDGEMENT. This work was supported by the Louisiana chapter of the American Heart Association (MRP) and NIH RO1 HD31885 (MJSM).

**Received 28 August 1995; accepted in revised form 9 October 1995**

## References

[PDF_00573] Baylis C., Mitruka B., Deng A. (1992). Chronic blockade of nitric oxide synthesis in the rat produces systemic hypertension and glomerular damage.. J Clin Invest.

[PDF_00533] Davidson D., Eldemerdash A. (1990). Endothelium-derived relaxing factor: presence in pulmonary and systemic arteries of the newborn guinea pig.. Pediatr Res.

[PDF_00554] Fairweather-Tait S., Fox T., Wharf S. G., Eagles J. (1995). The bioavailability of iron in different weaning foods and the enhancing effect of a fruit drink containing ascorbic acid.. Pediatr Res.

[PDF_00600] Fineman J. R., Heymann M. A., Soifer S. J. (1991). N omega-nitro-L-arginine attenuates endothelium-dependent pulmonary vasodilation in lambs.. Am J Physiol.

[PDF_00603] Frank L. (1991). Developmental aspects of experimental pulmonary oxygen toxicity.. Free Radic Biol Med.

[PDF_00611] Frank L., Sosenko I. R. (1991). Failure of premature rabbits to increase antioxidant enzymes during hyperoxic exposure: increased susceptibility to pulmonary oxygen toxicity compared with term rabbits.. Pediatr Res.

[PDF_00589] Gasser R. F., Shigihara S., Shimada K. (1994). Three-dimensional development of the facial nerve path through the ear region in human embryos.. Ann Otol Rhinol Laryngol.

[PDF_00566] Grisham M. B., Specian R. D., Zimmerman T. E. (1994). Effects of nitric oxide synthase inhibition on the pathophysiology observed in a model of chronic granulomatous colitis.. J Pharmacol Exp Ther.

[PDF_00578] Holmes R. S., Masters C. J. (1970). Epigenetic interconversions of the multiple forms of mouse liver catalase.. FEBS Lett.

[PDF_00530] Holzmann S. (1982). Endothelium-induced relaxation by acetylcholine associated with larger rises in cyclic GMP in coronary arterial strips.. J Cyclic Nucleotide Res.

[PDF_00597] Ignarro L. J., Burke T. M., Wood K. S., Wolin M. S., Kadowitz P. J. (1984). Association between cyclic GMP accumulation and acetylcholine-elicited relaxation of bovine intrapulmonary artery.. J Pharmacol Exp Ther.

[PDF_00618] Kourembanas S., McQuillan L. P., Leung G. K., Faller D. V. (1993). Nitric oxide regulates the expression of vasoconstrictors and growth factors by vascular endothelium under both normoxia and hypoxia.. J Clin Invest.

[PDF_00576] McCord J. M., Fridovich I. (1969). Superoxide dismutase. An enzymic function for erythrocuprein (hemocuprein).. J Biol Chem.

[PDF_00563] Miller M. J., Sadowska-Krowicka H., Chotinaruemol S., Kakkis J. L., Clark D. A. (1993). Amelioration of chronic ileitis by nitric oxide synthase inhibition.. J Pharmacol Exp Ther.

[PDF_00580] Paglia D. E., Valentine W. N. (1967). Studies on the quantitative and qualitative characterization of erythrocyte glutathione peroxidase.. J Lab Clin Med.

[PDF_00586] Richards G. M. (1974). Modifications of the diphenylamine reaction giving increased sensitivity and simplicity in the estimation of DNA.. Anal Biochem.

[PDF_00538] Rossaint R., Falke K. J., López F., Slama K., Pison U., Zapol W. M. (1993). Inhaled nitric oxide for the adult respiratory distress syndrome.. N Engl J Med.

[PDF_00583] Schacterle G. R., Pollack R. L. (1973). A simplified method for the quantitative assay of small amounts of protein in biologic material.. Anal Biochem.

[PDF_00557] Voelker C. A., Miller M. J., Zhang X. J., Eloby-Childress S., Clark D. A., Pierce M. R. (1995). Perinatal nitric oxide synthase inhibition retards neonatal growth by inducing hypertrophic pyloric stenosis in rats.. Pediatr Res.

[PDF_00546] Zweier J. L., Duke S. S., Kuppusamy P., Sylvester J. T., Gabrielson E. W. (1989). Electron paramagnetic resonance evidence that cellular oxygen toxicity is caused by the generation of superoxide and hydroxyl free radicals.. FEBS Lett.

